# Supplementary material for: Analysis of PPARγ Signaling Activity in Psoriasis
Source: Int J Mol Sci. 2021 Aug 10;22(16):8603. doi: 10.3390/ijms22168603 (PMC8395241; doi:10.3390/ijms22168603)
Supplement: Supplementary file 1 [file ijms-22-08603-s001.zip › Supplemental materials_Analysis of PPARg signaling activity in psoriasis/Pathway models/Models images and html files/Differentiation of psoriatic T cells/6196.html]

IFNGR1


# Protein IFNGR1

|  |  |
| --- | --- |
| URN | urn:agi-llid:3459 |
| Total Entities | 0 |
| Connectivity | 593 |
| Name | IFNGR1 |
| Description | interferon gamma receptor 1 |
| Notes | This gene (IFNGR1) encodes the ligand-binding chain (alpha) of the gamma interferon receptor. Human interferon-gamma receptor is a heterodimer of IFNGR1 and IFNGR2. A genetic variation in IFNGR1 is associated with susceptibility to Helicobacter pylori infection. In addition, defects in IFNGR1 are a cause of mendelian susceptibility to mycobacterial disease, also known as familial disseminated atypical mycobacterial infection. [provided by RefSeq, Jul 2008] |
| Primary Cell Localization | Plasma membrane |
| Class | Receptor |
| ObjectType | Protein |

---

|  |  |
| --- | --- |
| Pathway | Natural Killer Cell Activation |
|  | T-Cell Cytotoxic Mediated Cell Death |
|  | Th1-Cell Differentiation |
|  | Proplatelet Maturation |
|  | Macrophage M1 Lineage |
|  | AHR Signaling in M1 Macrophages Function |
|  | Proteins Involved in Atherosclerosis |
|  | Th1-Cells Activation and Proliferation in Atherosclerosis |
|  | Lymphocyte Mediated Myocardial Injury in Myocarditis |
|  | SOCS in Negative Feedback Regulation in Myocarditis |
|  | Atopic Dermatitis |
|  | T-Cells Differentiation Block in Psoriasis |
|  | IFN-gamma/TNF-alpha Mediated Cell Proliferation in Psoriasis |
|  | Th1-Cell Function in Systemic Lupus Erythematosus |
|  | Vitiligo |
|  | T-Cell Cytotoxic Response against Melanocytes in Vitiligo |
|  | Toll-like Receptors in beta-Cell |
|  | beta-Cell Destruction through Cytokines in Diabetes Mellitus |
|  | beta-Cell Destruction in Diabetes Mellitus Type 1 |
|  | Nitric Oxide Effects on beta-Cell |
|  | Hashimoto's Thyroiditis |
|  | CD4+T-Cell Response in Celiac Disease |
|  | Metastatic Colorectal Cancer |
|  | Th1-Cell Activation in Crohn's Disease |
|  | Keratinocyte Activation in Psoriatic Arthritis |
|  | IFNG -> ARRB1/STAT1 Signaling |
|  | CSF2 -> NF-kB Expression Targets |
|  | Clozapine Induced Granulocytopenia |
|  | Myeloid Derived Suppressor Cells in Cancer Immune Escape |
|  | IDO1 in Cancer Immune Escape |
|  | IFNG/IFNR Expression Targets |
|  | Ras-GAP Regulation Signaling |
|  | TNF -> NF-kB Expression Targets |
|  | IL1B Expression Targets |
|  | PPAR Psoriasis |
|  | IFN signaling in alveolar cells |
|  | 1\_Differentiation of psoriatic T cells |
|  | 4\_Inflammatory mediators inhibit GATA signaling in erythropoiesis |
|  | 1\_Antigen-presenting cells promote islet dysfunction and immune system activation in T1D |
|  | 3\_Islet beta-cell destruction in T1D |
|  | Differentiation of psoriatic T cells |

---

|  |  |
| --- | --- |
| Group | Genes Associated with Systemic Lupus Erythematosus |

---

|  |  |
| --- | --- |
| MedScan ID | 3459 |

---

|  |  |
| --- | --- |
| LocusLink ID | 3459 |
|  | 15979 |
|  | 116465 |

---

|  |  |
| --- | --- |
| Alias | Ifgr |
|  | RP3-503F13.3 |
|  | OTTHUMP00000017286 |
|  | OTTHUMP00000017285 |
|  | Nktar |
|  | IFN gamma R1 |
|  | CDw119 |
|  | interferon gamma receptor 1 |
|  | IFNGR1 receptor |
|  | antiviral protein type 2 |
|  | IFN gamma receptor 1 |
|  | CD119 |
|  | INGR\_HUMAN |
|  | IFNGR1 |
|  | IFN-gamma-R-alpha |
|  | interferon gamma receptor I |
|  | receptor IFNGR1 |
|  | IFNGR I |
|  | CD119 antigen |
|  | FLJ45734 |
|  | Interferon-gamma receptor alpha chain |
|  | IFN gamma receptor I |
|  | immune interferon receptor 1 |
|  | AVP2 |
|  | gamma interferon receptor 1 |
|  | LOC360301 |
|  | IFNGR |
|  | IMD27A |
|  | IMD27B |
|  | AVP, type 2 |
|  | IFN-gamma receptor 1 |
|  | IFN-gamma-R1 |
|  | antiviral protein, type 2 |
|  | IFN-gammaR |
|  | INF-g receptor |
|  | interferon gamma receptor alpha-chain |
|  | Immune interferon receptors |
|  | interferon-gamma receptor 1 |
|  | INGR |
|  | receptor for immune interferon |
|  | ifn-gamma r |
|  | INF-g receptors |
|  | interferon gamma receptor |

---

|  |  |
| --- | --- |
| GO ID | 0019955 |
|  | 0004896 |
|  | 0004906 |
|  | 0048143 |
|  | 0019221 |
|  | 0060333 |
|  | 0001774 |
|  | 1900222 |
|  | 1902004 |
|  | 0010628 |
|  | 1904469 |
|  | 0060334 |
|  | 0009615 |
|  | 0007165 |
|  | 0005887 |
|  | 0005886 |
|  | 0051607 |
|  | 1904783 |
|  | 0030425 |
|  | 0005783 |
|  | 0016021 |
|  | 0016020 |
|  | 0014069 |
|  | 0031982 |
|  | 0042015 |
|  | 0004872 |

---

|  |  |
| --- | --- |
| KEGG ID | hsa:3459 |
|  | mmu:15979 |
|  | rno:116465 |

---

|  |  |
| --- | --- |
| Organism | Homo sapiens {Organism urn:agi-taxid:9606} |
|  | Mus musculus {Organism urn:agi-taxid:10090} |
|  | Rattus norvegicus {Organism urn:agi-taxid:10116} |
|  | Mus musculus |
|  | Homo sapiens |
|  | Rattus norvegicus |

---

|  |  |
| --- | --- |
| Mouse chromosome position | 10 8.49 cM |
|  | 10 15.0 cM |

---

|  |  |
| --- | --- |
| OMIM ID | 107470 |
|  | 209950 |
|  | 600263 |
|  | 610424 |
|  | 615978 |
|  | 607948 |

---

|  |  |
| --- | --- |
| Rat chromosome position | 1p12 |

---

|  |  |
| --- | --- |
| Hugo ID | 5439 |
|  | HGNC:5439 |

---

|  |  |
| --- | --- |
| Human chromosome position | 6q23.3 |

---

|  |  |
| --- | --- |
| Swiss-Prot Accession | A0A0S2Z3Y2 |
|  | P15260 |
|  | P15260.1 |
|  | P15261 |
|  | P15261.3 |
|  | Q9QZ62 |
|  | B4DFT7 |
|  | E1P587 |
|  | Q53Y96 |
|  | Q91Y85 |
|  | F5H5M7 |
|  | Q14936 |
|  | Q53GX8 |
|  | Q5TFD1 |
|  | Q7Z687 |
|  | Q9BY69 |
|  | Q3TW49 |
|  | Q3U8T2 |
|  | Q540M5 |
|  | P70599 |
|  | Q6P6T3 |

---

|  |  |
| --- | --- |
| PIR ID | A31555 |
|  | A34368 |

---

|  |  |
| --- | --- |
| GenBank ID | NC\_000006 |
|  | XM\_011535793 |
|  | XP\_011534095 |
|  | NM\_000416 |
|  | NP\_000407 |
|  | NM\_001363526 |
|  | NP\_001350455 |
|  | NM\_001363527 |
|  | NP\_001350456 |
|  | XM\_006715470 |
|  | XP\_006715533 |
|  | XM\_011535794 |
|  | XP\_011534096 |
|  | NG\_007394 |
|  | AH006710 |
|  | AAC52064 |
|  | AJ490331 |
|  | CAD35933 |
|  | AJ490332 |
|  | CAD35932 |
|  | AL050337 |
|  | AY594694 |
|  | AAS89302 |
|  | CH471051 |
|  | EAW47931 |
|  | EAW47932 |
|  | HQ670233 |
|  | ADR72811 |
|  | HQ670234 |
|  | ADR72812 |
|  | HQ670235 |
|  | ADR72813 |
|  | HQ670236 |
|  | ADR72814 |
|  | HQ670237 |
|  | ADR72815 |
|  | AF056979 |
|  | AAK30623 |
|  | AK127636 |
|  | AK222803 |
|  | BAD96523 |
|  | AK294252 |
|  | BAG57548 |
|  | AK297052 |
|  | BAG59578 |
|  | AK312674 |
|  | BAG35556 |
|  | BC005333 |
|  | AAH05333 |
|  | BT006814 |
|  | AAP35460 |
|  | BU674317 |
|  | DC349115 |
|  | EF173879 |
|  | ABM53152 |
|  | EF535103 |
|  | ABQ00413 |
|  | J03143 |
|  | AAA52731 |
|  | KU178144 |
|  | ALQ33602 |
|  | KU178145 |
|  | ALQ33603 |
|  | P15260 |
|  | NC\_000076 |
|  | NM\_010511 |
|  | NP\_034641 |
|  | AC153862 |
|  | CH466562 |
|  | EDL03452 |
|  | U05960 |
|  | AAA80980 |
|  | AF128216 |
|  | AAF22557 |
|  | AF128217 |
|  | AAF22558 |
|  | AK087525 |
|  | AK150503 |
|  | BAE29616 |
|  | AK151079 |
|  | BAE30094 |
|  | AK152085 |
|  | BAE30935 |
|  | AK152384 |
|  | BAE31172 |
|  | AK153084 |
|  | BAE31709 |
|  | AK153347 |
|  | BAE31923 |
|  | AK159359 |
|  | BAE35017 |
|  | AK159660 |
|  | BAE35269 |
|  | AK159839 |
|  | BAE35417 |
|  | AK169593 |
|  | BAE41245 |
|  | AY157836 |
|  | AAO21311 |
|  | AY157837 |
|  | AAO21312 |
|  | BC132599 |
|  | AAI32600 |
|  | BC138617 |
|  | AAI38618 |
|  | BU700524 |
|  | BY309097 |
|  | BY754033 |
|  | J05265 |
|  | AAA39178 |
|  | M25764 |
|  | AAA39177 |
|  | M26711 |
|  | AAA37896 |
|  | M28233 |
|  | AAA37898 |
|  | M28995 |
|  | AAA37895 |
|  | P15261 |
|  | NC\_005100 |
|  | NM\_053783 |
|  | NP\_446235 |
|  | AC\_000069 |
|  | AAHX01001705 |
|  | AC116231 |
|  | CH473994 |
|  | EDL93784 |
|  | AF201901 |
|  | AAF08977 |
|  | BC062039 |
|  | AAH62039 |
|  | U68272 |
|  | AAB17055 |
|  | XP\_006715534 |
|  | XM\_006715471 |
|  | XP\_016866316 |
|  | XM\_017010827 |
|  | U19247 |
|  | AAHY01083833 |
|  | ABBA01025818 |
|  | XP\_005267015 |
|  | U19242 |
|  | U19241 |
|  | U19244 |
|  | U19243 |
|  | U19246 |
|  | U19245 |
|  | XP\_005267016 |
|  | AABR06000779 |
|  | AC\_000032 |
|  | AC\_000138 |
|  | XM\_005266959 |
|  | XM\_005266958 |
|  | AMYH02014569 |
|  | AMYH02014568 |
|  | NC\_018917 |
|  | CAB53062 |
|  | CAI21592 |
|  | CAI21593 |
|  | NT\_025741 |
|  | NW\_001838990 |
|  | NW\_923184 |
|  | AC\_000049 |
|  | CR594485 |
|  | CR601864 |
|  | CR624388 |
|  | CR625186 |
|  | Q14936 |
|  | Q53GX8 |
|  | Q5TFD1 |
|  | Q7Z687 |
|  | Q9BY69 |
|  | NT\_039492 |
|  | NW\_001030408 |
|  | Q3TW49 |
|  | Q3U8T2 |
|  | Q540M5 |
|  | Q91Y85 |
|  | NW\_001084746 |
|  | NW\_047544 |
|  | P70599 |
|  | Q6P6T3 |
|  | Q9QZ62 |

---

|  |  |
| --- | --- |
| Swiss-Prot ID | INGR1\_HUMAN |
|  | INGR1\_MOUSE |
|  | Q91Y85\_MOUSE |
|  | Q9QZ62\_RAT |
|  | Q53Y96\_HUMAN |

---

|  |  |
| --- | --- |
| Cell Localization | Cell membrane |
|  | Membrane |
|  | Plasma membrane |

---

|  |  |
| --- | --- |
| Ensembl ID | ENSG00000027697 |
|  | ENSP00000356713.4 |
|  | ENST00000367739.8 |
|  | ENSP00000494069.1 |
|  | ENST00000646898.1 |
|  | ENSMUSG00000020009 |
|  | ENSMUSP00000020188.6 |
|  | ENSMUST00000020188.12 |
|  | ENSRNOG00000012074 |
|  | ENSRNOP00000016286.4 |
|  | ENSRNOT00000016286.6 |
|  | ENST00000646036.1 |
|  | ENST00000644894.1 |
|  | ENSP00000496387.1 |
|  | ENSP00000495272.1 |
|  | ENSRNOP00000016286 |
|  | ENSMUST00000020188 |
|  | ENSRNOT00000016286 |
|  | ENSP00000443282 |
|  | ENSP00000356713 |
|  | ENST00000543628 |
|  | ENST00000367739 |
|  | ENSMUSP00000020188 |

---

|  |  |
| --- | --- |
| MGI ID | MGI:107655 |
|  | 107655 |

---

|  |  |
| --- | --- |
| RGD ID | 620570 |

---

|  |  |
| --- | --- |
| Unigene ID | Mm.549 |
|  | Hs.520414 |
|  | Rn.19927 |

---

|  |  |
| --- | --- |
| Homologene ID | 359 |

---

|  |  |
| --- | --- |
| Shape | Stick-vertex |

---

|  |  |
| --- | --- |
| IPI ID | IPI00323231 |
|  | IPI00010808 |
|  | IPI00129679 |
|  | IPI00327970 |
|  | IPI00514230 |
|  | IPI00514426 |

---
